# Pan-cancer analysis of chromothripsis-related gene expression patterns indicates an association with tumor immune and therapeutic agent responses

**DOI:** 10.3389/fonc.2023.1074955

**Published:** 2023-01-24

**Authors:** Qin Zhang, Lujie Yang, He Xiao, Zhaoqian Dang, Xunjie Kuang, Yanli Xiong, Jianwu Zhu, Zhou Huang, Mengxia Li

**Affiliations:** Cancer Center, Daping Hospital, Army Medical University, Chongqing, China

**Keywords:** chromothripsis, tumor immunity, drug resistance, prognosis, immune checkpoint inhibitors therapy

## Abstract

Chromothripsis is a catastrophic event involving numerous chromosomal rearrangements in confined genomic regions of one or a few chromosomes, causing complex effects on cells *via* the extensive structural variation. The development of whole-genome sequencing (WGS) has promoted great progress in exploring the mechanism and effect of chromothripsis. However, the gene expression characteristics of tumors undergone chromothripsis have not been well characterized. In this study, we found that the transcriptional profile of five tumor types experiencing chromothripsis is associated with an immune evasion phenotype. A gene set variation analysis (GSVA) was used to develop a CHP score, which is based on differentially expressed gene sets in the TCGA database, revealing that chromothripsis status in multiple cancers is consistent with an abnormal tumor immune microenvironment and immune cell cytotoxicity. Evaluation using four immunotherapy datasets uncovered the ability of the CHP score to predict immunotherapy response in diverse tumor types. In addition, the CHP score was found to be related to resistance against a variety of anti-tumor drugs, including anti-angiogenesis inhibitors and platinum genotoxins, while EGFR pathway inhibitors were found to possibly be sensitizers for high CHP score tumors. Univariate COX regression analysis indicated that the CHP score can be prognostic for several types of tumors. Our study has defined gene expression characteristics of tumors with chromothripsis, supporting the controversial link between chromothripsis and tumor immunity. We also describe the potential value of the CHP score in predicting the efficacy of immunotherapy and other treatments, elevating chromothripsis as a tool in clinical practice.

## Introduction

It is widely recognized that the mutations in the genetic material can result in cancers *via* activation of oncogenes or inactivation of tumor suppressors. This conventional paradigm believes the tumorigenesis is driven by the gradual accumulation of genomic damage and mutagenesis over time. However, a catastrophic event that entails extensive breakage and random rearrangements on focal chromosomes, termed chromothripsis, has been argued to be a novel targeted event that initiates a considerable number of human cancers (1, 2). In a single chromothripsis incident, hundreds of DNA breaks can occur in a short time. Consequently, overwhelmed or erroneous DNA repair processes lead to oncogenic fusion/amplification and loss of tumor suppressor genes, seeding the carcinogenic transformation of normal cells (3). Furthermore, this chromosome rearrangement phenomenon is considered to be one of the drivers of tumor evolution and is related to a dismal prognosis for cancer patients (4–6). Recently, structural variation (SV) viewed by whole-genome sequencing (WGS) has revealed that the actual prevalence of chromothripsis is much higher than estimated from earlier studies (7).

Much of the prior work on chromothripsis had focused on the complex variation of the resulting chromosome structures; however, great progress has been made since in exploring the mechanism and the biological and pathological effects of chromothripsis. Most notably, studies of micronuclei have revealed novel links between chromothripsis and the malignant biological behavior of tumors. In situations of drug selection, continuous genomic breakage-fusion-bridges cycles produce circular extrachromosomal DNA (ecDNA) that is encapsulated in micronuclei that further support the occurrence of chromothripsis. The associated massive chromosomal rearrangements mediate enhanced drug resistance in turn (3, 8). Chromothripsis is also frequently observed in highly invasive tumor subtypes, such as pediatric medulloblastoma or late stage neuroblastoma, suggesting a connection to a more aggressive malignant phenotype as well (2, 5).

As recent evidence has revealed, chromothripsis is intricately associated with immunity. For example, numerous micronuclei are generated in tumors with chromothripsis, and the rupture of defective micronuclei envelopes can lead to activation of innate immunity through the DNA-activated cGAS-STING pathway (9–11). Additionally, genomic instability can drive immune surveillance (8), and chromosomal rearrangements can promote the production and presentation of neoantigens, which can in turn activate immune cells and the clearance of tumor cells in mesothelioma (12, 13). Alternatively, cells can limit the initiation of innate pro-inflammatory signals by inhibiting the release of DNA from micronuclei (14). A large sample study found that aneuploidy, a kind of SV, is associated with a neoantigen editing and presentation deficiency and can lead to a poor immunotherapy response (15). Thus, differing SVs may impart varying effects on immunity, emphasizing that the complex interaction between chromothripsis, genomic abnormalities and biological properties of tumors requires further clarification.

By analyzing the tumor gene expression profile (GEP) of tumors with chromothripsis, we can uncover novel and central features with respect to tumor immunity and drug tolerance. Here, we performed a pan-cancer analysis using the GEP of five tumor types and identified that chromothripsis is associated with tumor immune evasion. A chromothripsis related gene signature was then constructed and confirmed the correlation with immune escape and immunotherapy resistance. Furthermore, the CHP score was related to resistance against multiple anti-tumor drugs,e.g., mitosis and DNA replication inhibitors, whereas epidermal growth factor receptor (EGFR) pathway inhibitors were identified as potentially therapeutically beneficial for tumors with a high CHP score. In TCGA database, the CHP score was found to be a prognostic factor for many types of cancers, including breast invasive carcinoma (BRCA), endometrial carcinoma (UCEC), bladder urothelial carcinoma(BLCA), kidney renal clear cell carcinoma(KIRC), prostate adenocarcinoma(PRAD), and skin cutaneous melanoma(SKCM). Finally, by analyzing the mutational signature of the genome, we discovered that defects in homologous recombination (HR) or the APOBEC cytosine deaminase likely play important roles in the occurrence of chromothripsis in breast cancer. The study here has defined a gene expression signature of tumors with chromothripsis and provides critical insights into the implications of this event with regards to tumor cell behavior.

## Methods

### Data retrieving and preprocessing

Chromothripsis status of five types of cancer including BRCA, lung adenocarcinoma (LUAD), ovarian cancer (OV), stomach adenocarcinoma (STAD) and UCEC was retrieved from compbio.med.harvard.edu/chromothripsis. Of which, 68 samples indicated with “High confidence” or “Linked to high confidence” in any chromosome was categorized chromothripsis and 80 samples with “No” in all chromosomes was categorized as non-chromothripsis. Clinical information and RNA-seq expression profiles of TCGA Pan-Cancer Atlas Studies were obtained from www.cbioportal.org. A total number of 8912 tumor samples with RNA-seq expression profiles in the format of linear RSEM were finally included in pan-cancer analysis. Raw data of GSE194040, GSE91061, GSE11636 were downloaded from Gene Expression Omnibus (GEO). Raw count of RNA-seq and responsiveness to atezolizumab monotherapy in advanced bladder cancer was extracted from R package “IMvigor210CoreBiologies”. IC_50_ values in the natural logarithm of 268 anti-cancer drugs was downloaded from www.cancerrxgene.org/downloads/. The CEL files of Cancer Cell Line Encyclopedia(CCLE) cell lines was obtained from www.ebi.ac.uk/arrayexpress/experiments/E-MTAB-3610/. For RSEM and raw count data, variation stabilizing transformation (VST) implemented in *DESeq2* was applied prior to calculation of gene signatures and inference of abundance of infiltrated immune cells. For microarray profiled with affymetrix, function “rma” in R package *limma* was used to obtain expression matrix at probes level with default parameters. For raw data of Agilent, function “background Correct” and “normalize Between Arrays” were used to correct background and normalization. Function “neqc” was used for preprocessing data profiled with Illumina HumanHT-12 microarrays. The detailed sample size of each dataset used in this study were summarized in **Supplementary Table 1**.

### Identification of differentially expressed genes (DEGs) and development of CHP score

Given considerable difference in expression profiles among various cancers, the differentially expressed genes between chromothripsis (n=68) and non-chromothripsis (n=80) was identified regrading each cancer type as block by using *edgeR*. Gene set enrichment analysis (GSEA) was used to infer enriched pathways with gene rank based on log2 fold change and Molecular Signatures Database hallmark c5 gene sets. DEGs were filtrated with criteria: false discovery rate < 0.05 and absolute log2 fold change > 1. We took the advantage of gene set variation analysis (GSVA) by using DEGs listed in the **Supplementary Table 3**. The upregulated and downregulated genes generated two gene sets, which were referred to as “CHPsigP” and “CHPsigN”, respectively. The CHP score for each sample was calculated by taking the difference between the enrichment score of CHPsigP and CHPsigN which were both derived from the GSVA. Gene set variation analysis(GSVA) was utilized to VST normalized RNA-seq or microarray expression matrix with these two gene sets. The parameter mx.diff was set to TRUE in all GSVA analysis. The CHP score for each sample was calculated by subtracting enrichment score of CHPsigN from enrichment score of CHPsigP.

### Calculation of other gene signatures and immune cell infiltration

Cytotoxic T lymphocyte (CTL score) (16), interferon-gama(IFN-γ)-related T-cell-inflated gene signature (17), activated dendritic cells (DC score) (18), pan-fibroblast TGF-β response signature (FTR score) (19) was evaluated by calculating geometrical mean of expression of marker genes belonging to corresponding signature. The gene signature of Cytotoxic T lymphocyte was the marker genes of cell subcluster “Cytotoxic lymphocytes” originally presented in MCPcounter (16). For tumor immune dysfunction and exclusion (TIDE score), only dysfunction of T lymphocyte cells was considered and measured by calculating Pearson’s correlation coefficients between gene expression of marker genes and mean z statistic values of these marker genes from Cox regression in at least two cancer types as proposed by the original article (20). Abundance of 19 immune cell populations was evaluated with *ConsensusTME*. Calculation of all gene signatures and ConsensusTME were performed in VST normalized RNA-seq or microarray expression matrix.

### Analysis of genomic mutation in BRCA patients

TCGA dataset of BRCA patients with available mutation data was used for this analysis. The dataset was composed of 51 patients, which occurred chromothripsis events (N = 32) or non-chromothripsis events (N = 19). Analysis, summarize and visualize of mutation data were performed by maftools (https://github.com/PoisonAlien/maftools). Mutually exclusive or co-occurring set of genes could be detected using SomaticInteractions function, which performs pair-wise Fisher’s Exact test to detect such significant pair of genes. We also analyzed the differences in APOBEC enriched samples proportion and identified differentially altered genes in two cohorts. We took non-negative matrix factorization to decompose the matrix into n signatures, and shown comparison of similarities of detected mutational signatures against validated COSMIC mutational signatures.

### Statistical analysis

Kruskal-Wallis test with multiple comparisons or Wilcoxon test was used to evaluate differences in CHP score and other gene signatures among groups. In pan-cancer analysis, CHP score was categorized into high and low group with median value in each cancer type. Kaplan-Meier curves along with log rank test were used to evaluate difference of progression free survival(PFS) and overall survival(OS) between high and low CHP score groups. Univariate and multivariate Cox regression was used to evaluate hazard ratio and 95% confidence intervals for both continuous and categorized CHP score and other gene signatures for PFS and OS in pan-cancer analysis. In GSE194040, the difference in pathologically complete response (pCR) rate between high and low CHP score groups in each treatment arm was estimated by using Fisher’s exact probability. Univariate and multivariate logistic regression was used to evaluate the association of CHP score and other genes signatures and pCR. Receiver operating characteristic curve (ROC) analysis was conducted to evaluate the efficacy of CHP score to discriminate samples with chromothripsis from samples without chromothripsis or patients with responsiveness to immune checkpoint inhibitors (ICIs) from patients without response. All tests were two-sided. P value less than 0.05 was considered statistically significant.

## Results

### Chromothripsis is associated with a tumor evasion phenotype

**Figure 1:**
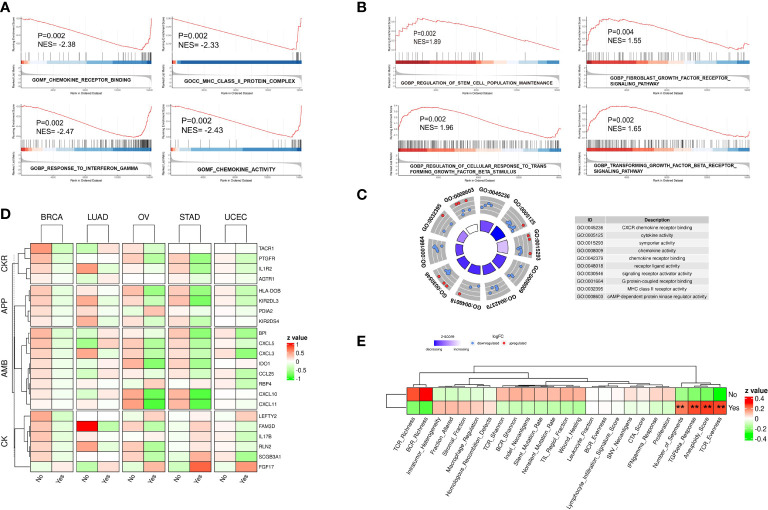
Gene expression profile of five types of cancer confirmed for chromothripsis status. **(A)** GSEA analysis reveals down-regulated pathways related to anti-tumor immunity in chromothripsis group. **(B)** GSEA analysis indicates up-regulated pathways related to tumor immune suppression in chromothripsis group. **(C)** GO analysis reveals enrichment of DEGs in cancer immune or protein interaction categories. **(D)** Expression of immune regulatory genes belonging to Chemokines (CK), Antimicrobials (AMB), Antigen processing and presentation (APP), and Cytokine receptors (CKR) in chromothripsis and non-chromothripsis groups of in five cancer types. **(E)** Immune cell activation signature in chromothripsis and non-chromothripsis groups. **p < 0.01.

Flow chart of this study was shown in **Supplementary Figure 1**. To identify the possible gene expression signature of tumors with chromothripsis, as defined by the criteria of the Pan-Cancer Analysis of Whole Genomes (7), patient material that underwent WGS and was confirmed for chromothripsis status was matched to their corresponding *in-situ* sequencing data in the TCGA database. After excluding tumor types with less than five chromothripsis positive samples, the expression data of the five remaining tumor types were collected (**Supplementary Table 1**): BRCA (n = 57), LUAD (n = 21), OV (n = 17), UCEC (n = 33) and STAD (n = 21). Patients were subsequently divided into chromothripsis (n=68) and non-chromothripsis (n=80) groups, and follow-up GSEA revealed that many pathways related to anti-tumor immunity, e.g., MHC class II complex, cytokine activity, and the IFN-γ response, were significantly underrepresented in the chromothripsis group (**Figure 1A**). Conversely, pathways related to tumor immunosuppression, including the transforming growth factor-β(TGF-β) and fibroblast growth factor receptor(FGFR) signal pathways, were significantly overrepresented in the chromothripsis group. In addition, we observed an overrepresentation of stemness maintenance in the chromothripsis group (**Figure 1B**) (**Supplementary Table 2**). Kyoto Encyclopedia of Genes and Genomes (KEGG) pathway enrichment analysis revealed that among the top 20 pathways, 7 pathways were related to tumor immunity and protein-protein interactions (**Supplementary Figure 2**).

Further data analysis identified 170 significantly DEGs between the two groups (**Supplementary Table 3**). Gene Ontology (GO) analysis showed that the DEGs, particularly the down-regulated genes, were significantly enriched in the chromothripsis group for biological processes related to chemokine receptor (CXCR) binding, cytokine activity, chemokine activity, chemokine receptor binding, and MHC class II receptor activity (all adjust p < 0.05) (**Figure 1C**). Consistent with this finding, the IMMPORT database classified the differentially expressed immunoregulatory genes into four groups: Chemokines (CK), Antimicrobials (AMB), Antigen processing and presentation (APP), and Cytokine receptors (CKR). Whereas most of the immunoregulatory genes were significantly decreased in the chromothripsis group, the expression of fibroblast growth factor 17 (FGF17) was significantly up-regulated (**Figure 1D**; **Supplementary Table 3**). By analyzing the activation of immune cells, we found that in the chromothripsis group: (i) T-cell receptor (TCR), B-cell receptor (BCR) richness was lower (P>0.05); (ii) fraction altered, HR defects, and intratumor heterogeneity were higher (P>0.05); and (iii) the aneuploid score, fragments number, TGF-β response and TCR evenness were significantly increased (P<0.05) (**Figure 1E**; **Supplementary Table 4**). These results indicate a likely association between chromothripsis and tumor immune evasion.

### Chromothripsis related gene signature indicates tumor immunosuppression

**Figure 2:**
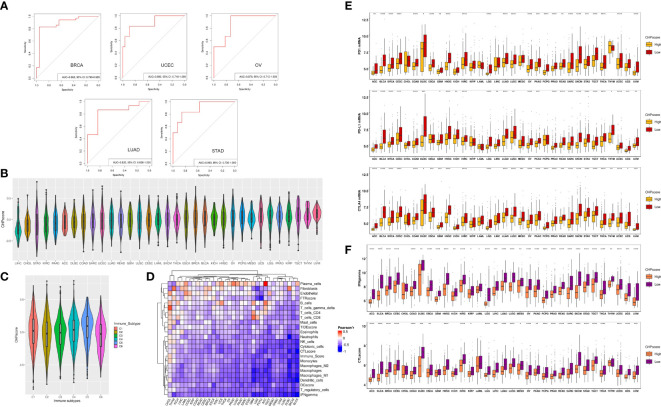
The construction of CHP score and the link with tumor immune evasion. **(A)** ROC curve indicates the ability of the CHP score to predict the occurrence of chromothripsis in five types of cancer. **(B)** The CHP score in all 33 types of cancer in the TCGA database. **(C)** The CHP score among all samples classified into C1, C2, C3, C4, and C5 groups. **(D)** The Pearson correlation coefficients of CHP score with the abundance of different immune cells and the immune signature in 33 types of cancer in TCGA database. **(E)** The expression of immune check-point factors in CHP score high and CHP score low groups. **(F)** Immune signature gene expression in CHP score high and CHP score low groups. *p < 0.05, **p < 0.01, ***p < 0.001, **** p < 0.0001.

To further validate the correlation between chromothripsis and tumor immunity, we used GSVA to develop a CHP score based on a chromothripsis related gene signature, which encompasses all DEGs between the chromothripsis and non-chromothripsis groups. The CHP score for each sample was calculated by subtracting the enrichment score of the downregulated gene sets from the upregulated gene sets (see Materials and Methods). Using ROC analysis, our studies show the ability of the CHP score to distinguish chromothripsis with area under the curve (AUC) values for BRCA (0.892,95%CI=0.799-0.985), UCEC (0.892,95%CI=0.799-0.985), LUAD (0.833,95%CI=0.636-1), OV (0.889,95%CI=0.718-1), and STAD (0.893,95%CI=0.736-1) (**Figure 2A**).

We next evaluated the CHP score using all 33 tumor types (n=8912) in the TCGA. First, uveal melanoma (UVM) was found to have the highest CHP score, whereas hepatocellular carcinoma (LIHC) had the lowest CHP score (**Figure 2B**). Second, Thorsson et al. identified six immune subtypes by pan-cancer analysis (21). We observed that type C5 (immunologically quiet) had the highest CHP score, while C2 (IFN-γ dominant) and C6 (TGF-β dominant) have the two lowest CHP scores (**Figure 2C**). Additionally, we found that the CHP score was negatively correlated with the IFN-γ response, Immune score, DC score, and CTL score and is associated with a low abundance of DC cells, NK cells, and monocytes in all 33 tumors. In partial tumors, such as BRCA and THYM, the CHP score was positively correlated with the abundance of plasma cells, fibroblasts and endothelial cells (**Figure 2D**).

We classified tumors into high and low CHP score groups for each cancer type according to the median value of the CHP score. Notably, the expression of immune checkpoint genes, including programmed cell death protein 1 (PD-1), programmed cell death ligand 1 (PD-L1), and Cytotoxic T-lymphocyte antigen number 4 (CTLA-4), was significantly lower in tumors with a high CHP score in nearly all cancer types (i.e., in 25, 27 or 28 of 33, respectively) (**Figure 2E**), as was the CTL score and the IFN-γ signature (**Figure 2F**) (**Supplementary Table 5**). Altogether, the results indicate that the CHP score reflects a tumor immunosuppression phenotype, which is characterized by a deficiency in immune cell infiltration and cytotoxicity.

### CHP score is related to poor immunotherapy response

**Figure 3:**
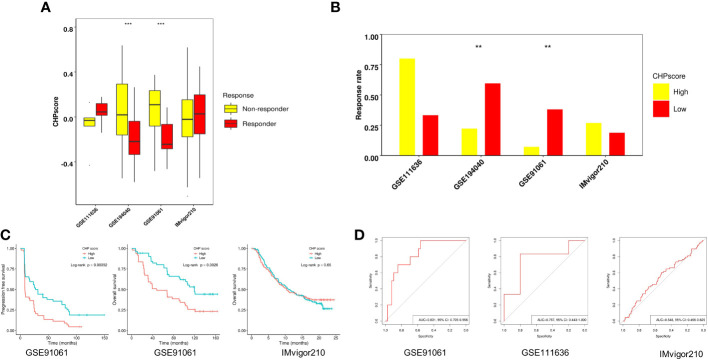
Role of CHP score in predicting the immunotherapy response in four GEO datasets. **(A)** The CHP score in responder and non-responder groups in four GEO datasets. **(B)** Respond rate in CHP score high and CHP score low groups in four GEO immunotherapy datasets. **(C)** K-M plot showing the PFS or OS of patients in two GEO immunotherapy datasets. **(D)** ROC curve indicates the ability of the CHP score to predict the immunotherapy response in three GEO datasets.

The efficacy of immunotherapy can be affected by many factors including tumor immune cell infiltration and/or activation, the expression of immune checkpoints, and tumor mutational burden. Our results revealed a relationship between chromothripsis and tumor immune evasion. We therefore used four GEO datasets, i.e., GSE91061, GSE111636, IMvigor210, and the immunotherapy arm of GSE194040, to validate the association of the CHP score with the immunotherapy response. The CHP score was found to be significantly higher in the non-responder group in comparison to the responder group in the GSE91061 and GSE194040 datasets (**Figure 3A**; **Supplementary Table 6**, **7**). Moreover, the immunotherapy response rate was significantly lower in the high CHP score group in these two datasets (**Figure 3B**). Survival analysis showed that the CHP score can reveal the prognosis of patients in the GSE91061 dataset, where patients with higher scores had lower PFS (log-rank P < 0.001) and OS (log-rank P= 0.0026). However, this relationship between the CHP score and survival did not hold true in the IMvigor210 dataset (log-rank P > 0.05) (**Figure 3C**). ROC analysis, which was used to verify the above observations, supported the conclusion that the CHP score predicts immunotherapy effectiveness. Specifically, the results indicate that the CHP score achieves good performance in both the GSE91061 (AUC=0.831,95%CI=0.705-0.956) and the GSE111636 (AUC=0.767,95%CI=0.443-1) dataset, but not in the IMvigor210 dataset (AUC=0.548,95%CI=0.466-0.625) (**Figure 3D**). These findings support the link between chromothripsis and an impaired response to immune checkpoint blockades and indicate the potential value of the CHP score in predicting the efficiency of immunotherapy in LUAD and BRCA cancers.

### CHP score is related to resistance to multiple anti-tumor agents

**Figure 4:**
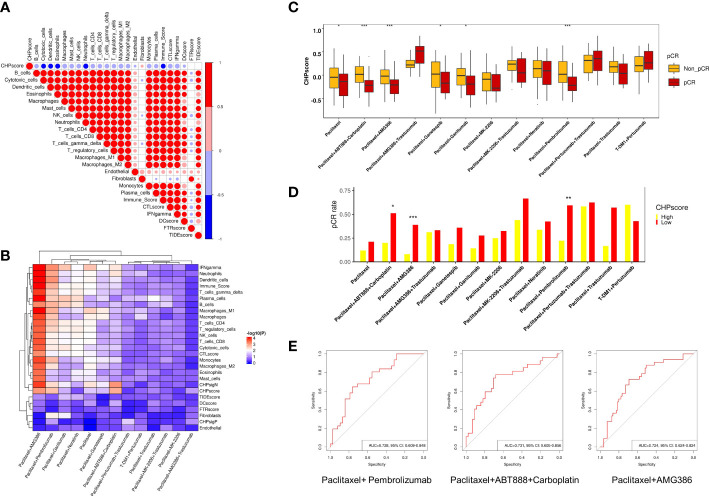
Role of CHP score in predicting the response to multiple-therapies in GSE194040 dataset. **(A)** Correlation coefficients between CHP score and the abundance of various immune cells or the expression of the immune signature in the GSE194040 dataset. **(B)** Correlation coefficients between the CHP score or specific immune signatures with responses to different treatments in the GSE194040 dataset. **(C)** The pCR rate of different treatments in CHP score high and CHP score low groups in the GSE194040 dataset. **(D)** The CHP score for different treatments between responder and non-responder groups. **(E)** ROC curves show the ability of the CHP score to predict the response to Paclitaxel+ Pembrolizumab, Paclitaxel+ABT888+Carboplatin, and Paclitaxel+AMG386. *p < 0.05, **p < 0.01, ***p < 0.001.

Many studies have revealed a close relationship between chromothripsis and therapeutic resistance. To explore the predictive ability of the CHP score for other treatments in the setting of neoadjuvant therapy, we used the GSE194040 dataset, which contains information on 988 breast cancer patients who received 13 arms of neoadjuvants (**Supplementary Table 1**). First, we analyzed the immune cells infiltration and immune activation signatures(see Materials and Methods) of all patients. Similar to the results of the TCGA database, the CHP score is negatively correlated with the abundance of most immune cells, including cytotoxic cells, dendritic cells, and CD4^+^, CD8^+^ T cells, but positively correlated for the abundance of fibroblasts (**Figure 4A**). Next, logistic regression analysis was performed to determine the correlation between various immune signatures and the pCR. Like some known immune signatures (e.g., IFN-γ, immune score and T cell abundance), the CHP score is significantly associated with the response to Paclitaxel+Pembrolizumab treatment (R=-3.34, P=0.002). Furthermore, the CHP score can predict the response to Paclitaxel (R=-1.62, P=0.03), Paclitaxel+AMG386 (R=-3.25, P<0.001), Paclitaxel+ABT888+Carboplatin (R=-3.71, P=0.001), Paclitaxel+Ganetespib (R=-1.62, P=0.038), and Paclitaxel+Ganitumab (R=-1.75, P=0.05) (**Figure 4B**) (**Supplementary Table 8**). The CHP score was higher in the non-pCR group of patients that received one of the above six treatments (**Figure 4C**). After correcting for hormone receptor and human epidermal growth factor receptor 2 (HER-2) factors, multivariate logistic regression analysis validated that the CHP score was significantly correlated with a poor response to Paclitaxel+Pembrolizumab (R=-2.84, P=0.012), Paclitaxel+AMG386 (R=-2.56, P=0.014), and Paclitaxel+ABT888+Carboplatin (R=-3.16, P=0.011) (**Supplementary Table 9**, **Supplementary Figure 2**). Meanwhile, the pCR of patients with a high CHP score was significantly reduced in these three treatment groups (all p < 0.05) (**Figure 4D**, **Supplementary Table 10**). ROC analysis confirmed the predictive power of the CHP score with regards to therapies involving Paclitaxel+Pembrolizumab (AUC=0.728,95%CI=0.609-0.848), Paclitaxel+AMG386 (AUC=0.731,95%CI=0.605-0.856), or Paclitaxel+ABT888+Carboplatin (AUC=0.724,95%CI=0.624-0.824) (**Figure 4E**). The results collectively indicate that the CHP score not only associates with immunotherapy responses but with tolerance to a range of anti-tumor treatment regimens, particularly anti-angiogenic inhibitors and platinum-based compounds in cases of breast cancer.

### EGFR pathway inhibitors are sensitizing drugs for tumors with high CHP score

**Figure 5:**
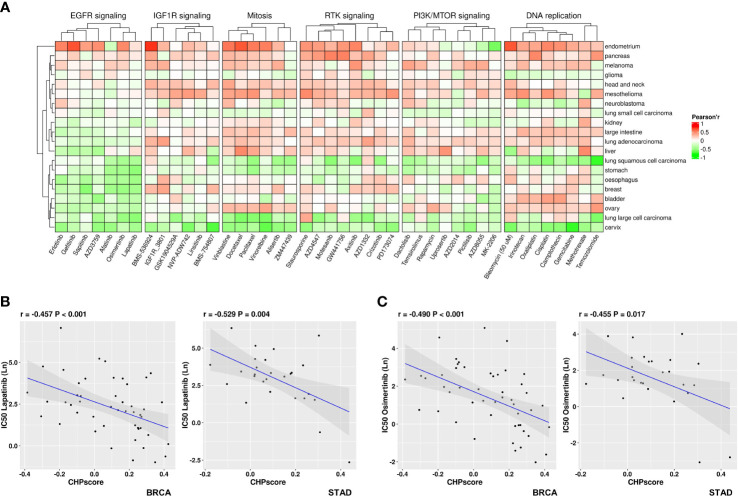
Sensitivity of tumors with high CHP score to different drugs in the GDSC database. **(A)** Correlation coefficient between the CHP score and the IC50 values of specified drugs in different pathways or targets. **(B)** Correlation between the CHP score and the IC50 values of Lapatinib in breast cancer (left) or gastric cancer (right). **(C)** Correlation between the CHP score and the IC50 values of Osimertinib in breast cancer (left) or gastric cancer (right).

The Genomics of Drug Sensitivity in Cancer (GDSC) database was next used to screen for potentially sensitizing drugs for tumors with a high CHP score. After excluding down-regulated genes that are mostly related to tumor immune regulation, the enrichment score of CHPsigP was used to identify chromothripsis status instead of the CHP score for each cell line. The Pearson correlation coefficient was then used to determine sensitivity to different drugs, where a significant negative correlation between the CHPsigP score and the half maximal inhibitory concentration (IC_50_) indicates a higher drug sensitivity. As shown in **Figure 5A**, the CHPsigP score is positively correlated with the IC_50_ of insulin like growth factor 1 receptor (IGF1R), receptor tyrosine kinase(RTK), DNA replication, and PI3K-mTOR pathway inhibitors, indicative of a tolerance against these compounds in CHPsigP high tumors. Notably, there was a negative correlation between the CHPsigP score and the IC_50_ of EGFR signal pathway inhibitors, suggesting that such drugs might be effective sensitizers against CHPsigP high tumors. Indeed, as revealed in ANOVA analysis, Pearson coefficients of drugs targeting EGFR signaling were significantly lower than those of drugs targeting other pathways (**Supplementary Table 11**). Furthermore, the IC_50_ of lapatinib, a HER-2 and EGFR tyrosine kinase inhibitor, is significantly negatively correlated with the CHPsigP score in breast (r=-0.457, P<0.001) and gastric cancer (r=-0.529, P=0.004) (**Figure 5B**). A similar relationship was also seen for osimertinib in breast (r=-0.490, P<0.001) and gastric cancer (r=-0.455, P=0.017) (**Figure 5C**).

### CHP score is a prognostic factor for the outcomes of multiple-cancer types

**Figure 6:**
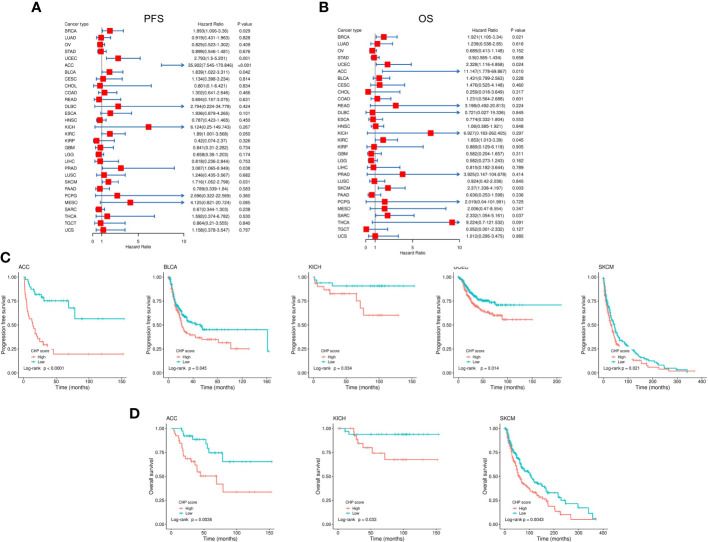
Evaluation of the correlation between CHP score and survival in 32 types of cancers. **(A-B)** Correlation between the CHP score and PFS **(A)** and OS **(B)** of 32 tumor types in the TCGA Database. **(C)** Kaplan-Meier curves of PFS in ACC, BLCA, KICH, UCEC, and SKCM. Patients were classified into CHP score high or low groups according to the median value of the CHP score. **(D)** Kaplan-Meier curves of OS in ACC, KICH, and SKCM, after patients were classified into CHP score high or low groups.

Many studies have shown that chromothripsis is associated with poor clinical outcomes (7, 22). Univariate cox regression uncovered that the CHP score was significantly correlated with worse PFS in BRCA (HR=1.89,95%CI=1.07-3.36,P=0.029), UCEC (HR=2.79,95%CI=1.5-5.2,P=0.0012), ACC (HR=35.9,95%CI=7.54-170.86,P<0.001), BLCA (HR=1.84,95%CI=1.02-3.31,P=0.042), KIRC (HR=1.89,95%CI=1-3.57,P=0.049), PRAD(HR=3.1,95%CI=1.07-8.95,P=0.038), and SKCM (HR=1.72,95%CI=1.05-2.8,P=0.031) (**Figure 6A**) and with worse OS in BRCA (HR=1.92,95%CI=1.10-3.34,P=0.021), UCEC (HR=2.33,95%CI=1.12-4.86,P=0.024), ACC (HR=11.15,95%CI=1.78-69.87,P=0.010), KIRC (HR=1.85,95%CI=1.01-3.39,P=0.045), SKCM (HR=2.37,95%CI=1.34-4.20,P=0.0031), and SARC (HR=2.33,95%CI=1.05-5.16,P=0.037) (**Figure 6B**). There was a significant difference in PFS between CHP score high and low tumors in cases of ACC, UCEC, BLCA, KICH, and SKCM, and in OS in ACC, KICH, and SKCM (**Figures 6C, D**). Thus, the CHP score is a prognostic factor for several types of cancers, and a high score is associated with a poor outcome in cases of ACC, UCEC, BLCA, KICH, and SKCM.

### HR defect and APOBEC are tightly link to chromothripsis in breast cancer

**Figure 7:**
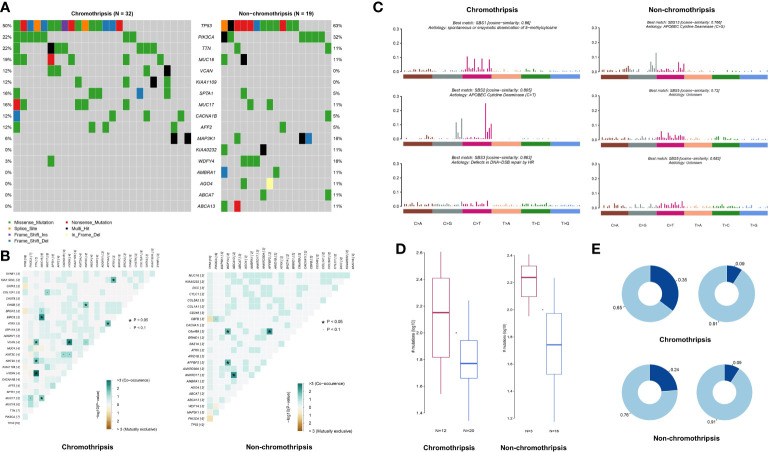
Genomic mutation characteristics of breast cancer in TCGA database. **(A)** Difference in mutation frequency between chromothripsis and non-chromothripsis groups in breast cancer. **(B)** Common mutation profiles of chromothripsis and non-chromothripsis groups in breast cancer. **(C)** Mutation signature of chromothripsis and non-chromothripsis groups in breast cancer. **(D)** Samples enriched or not for APOBEC in the chromothripsis and non-chromothripsis groups. **(E)** TCW motif burden in samples enriched or not for APOBEC in the chromothripsis and non-chromothripsis groups.

Genome mutation profiles were used to analyze the potential mechanisms underlying chromothripsis. Due to the limited numbers of chromothripsis positive samples in most cancer type collections, we were restricted to analyzing genomic mutation features in breast cancer only. As shown by **Figure 7A**, compared with the non-chromothripsis group (n=19), tumors with chromothripsis (n=32) have a higher frequency of mutations in the VCAN and KIAA1109 genes, which are related to immunotherapeutic efficiency and prognosis in several types of cancer (23, 24). Somatic Interactions analysis showed that the chromothripsis group has more co-mutation patterns than the non-chromothripsis group, possibly related to the hallmark chromosome rearrangements (**Figure 7B**). For instance, BRCA2-MUC16, PI3KCA-MUC17, ATRX-ATP1A4 et al. co-mutations were identified in chromothripsis group.

Signature of single base substitution (SBS) analysis was next performed, and the signatures of SBS1 (Spontaneous or enzymatic deamination of 5-methylcytosine), SBS2 (C > T mutation mediate by APOBEC cytidine deaminase), and SBS3 (Defects in DNA-Double strand break(DSB) repair by HR) were found in the chromothripsis group, whereas non-chromothripsis was enriched in SBS5 (Aetiology: Unknown) and SBS13 (C > G mutation mediate by APOBEC cytidine deaminase) (**Figure 7C**). Moreover, the proportion of APOBEC enriched samples is significantly higher in the chromothripsis group than in the non-chromothripsis group (37.5% vs. 15.8%) (**Figure 7D**), and the TCW motif burden of APOBEC enriched samples is also higher in the former group (0.35 vs. 0.24) (**Figure 7E**). These results reveal a specific mutation signature in breast cancers with chromothripsis and indicate that HR defect and APOBEC may play important roles in the occurrence and process of chromothripsis.

## Discussion

A great deal of evidence has shown that SVs associated with chromosome instability can have great impact on cells and are one of the main drivers of tumorigenesis (25). Chromothripsis is an extensive chromosomal rearrangement event, which typically has more complex effects than comparatively simple changes in a single chromosome segment, such as amplification, deletion or insertion. At present, chromothripsis is far less understood than other chromosome aberration events and has not been described as a potential biomarker to aid clinical treatment. Thus, using transcriptional profiling, we aimed to establish a comprehensive picture of gene expression characteristics for tumors with chromothripsis.

In analyzing gene expression profiles and evaluating tumor immune cell infiltration and activation, we report herein that chromothripsis is associated with a tumor immune evasion phenotype. Additionally, a decoded chromothripsis related gene signature, i.e., the CHP score, supports an immune escape phenotype of tumors with chromothripsis and correlates with a poor immunotherapy response. Although chromothripsis is a complex chromosomal rearrangement event that has neoantigenic potential, our results show an obvious suppression of immune cell infiltration and activation in chromothripsis tumor samples, echoing the complex relationship between aneuploid events, neoantigen editing/presentation and tumor immune escape seen in an earlier large sample study (15). A similar phenomenon is seen in colorectal cancer, where tumors with low immune cytolytic activity undergo more chromothriptic events and benefit little from immunotherapy (26). Notably, both the IFN-γ signature and immune cell activation were significantly reduced in tumors with chromothripsis, whereas the TGF-β signalling pathway was up-regulated. TGF-β plays a key role in cancer progression by reshaping the tumor immune microenvironment and promoting drug tolerance, angiogenesis and other cancer-beneficial effects (27). The low expression of immune checkpoint pathways might also contribute to the poor immunotherapy response. In rectal adenocarcinoma, chromothripsis on chromosome 9 results in the deletion and low expression of CD274 and PDCD1LG2 genes, which may play important roles in the poor response to immunotherapy (26).

Circular ecDNA provides another possible signal for the amplification/fusion of oncogenes and inactivation of suppressor genes, outcomes that can induce drug tolerance in tumors (3). In addition to immune escape, we observed that the CHP score correlates with resistance to multiple drugs, including anti-angiogenic inhibitors and the genotoxin platinum. Importantly, we found that EGFR pathway inhibitors were possibly beneficial for the treatment of tumors with a high CHP score. Seemingly consistent with this finding, chromothripsis is frequently detected in lung cancers driven by EGFR mutations (28), and mutation of EGFR and ERBB2 is associated with more rearrangement events in LUADs (29). A correlation between EGFR amplification and chromothripsis has also been detected in glioblastoma (30). However, in our study of breast cancer, no significant association has been observed in EGFR or ERBB2 mutations and chromothripsis, potentially because of technical limitations or insufficient sample size. More extensive analysis of the relationship between EGFR status and therapeutic targeting with chromothripsis is clearly warranted.

DNA repair processes are indispensable for the chromosome reassembly during chromothripsis, especially in the context of the widely-accepted micronucleus theory (31). TP53, a prominent tumor-suppressor gene, is closely associated with the level of aneuploidy and chromothripsis in many cancers, especially in several types of pediatric tumors (2, 32–35). Homologous recombination deficiency (HRD) is broadly defined in clinic, ranging from deleterious mutations in single HRR genes (i.e. BRCA1/2) to complex genomic scars (36). Our results found mutation of BRCA2 in chromotripsis group (**Figure 7B**).

Consistent with our result, a previous study demonstrated that BRCA2/p53-deficient mice enhance the probability for chromothripsis events (37). A high frequency of chromothripsis has also been detected in acute lymphoblastic leukemia with mutations in Ataxia-telangiectasia mutated (ATM), a key signaling kinase of HR (38). Indeed, a wide spectrum of DNA repair gene mutations are commonly associated with increased micronuclei and genomic rearrangements, including the Rad3-related protein (ATR), Nibrin (NBN) and RecQ protein-like 3 (RECQL3) (39–42). On the flip side, variation in DNA repair-related genes can be the result of chromothripsis, for instance, in colorectal cancer (43). Our results show that an HR defect signature is enriched in breast cancers with chromothripsis, suggesting that impairment in the faithful resolution of DSBs plays a prominent role in chromothripsis. Tumors with an HR defect may call upon more imprecise processes, such as non-homologous end joining (NHEJ) and/or microhomology-mediated end-joining (MMEJ), to repair DNA DSBs, resulting in abnormal processing or unwanted rearrangements. In fact, NHEJ or MMEJ signatures are frequently associated with abberant rearrangement events (44), and NHEJ is central to the formation of ecDNA (3) and appears to be the main repair mechanism for chromothripsis (45, 46).

Aberrant expression of the APOBEC family of proteins is an important driver of genome mutation and tumor evolution (47). Localized regions of hypermutation in certain cancer genomes, referred to as katagies, are thought to be the result of erroneous APOBEC activity (48). The accompanying C to T transition mutations are the outcome of cytosine deamination mediated by APOBEC enzymes that are enriched in the chromothripsis group, whereas uracil-initiated base excision repair mediated by uracil-DNA glycosylase (UNG) likely drives C to G transversion mutations in non-chromothripsis tumors (49, 50). Whether the APOBEC enzymes participate directly in chromothripsis and chromosome reshuffling warrants further investigation.

An increase in aneuploidy is related to poor prognosis of multiple tumor types (15, 51). Chromothripsis is associated with a worse prognosis in cases of multiple myeloma (4), neuroblastoma (5), and acute myeloid leukemia (6). This association was also suggested following two pan-cancer studies (7, 22). Multiple factors, including aggressiveness, tumor heterogeneity, and drug tolerance, can contribute to a poor outcome. A tumor-supportive immune microenvironment and dysfunctional immune cells can also lead to worse survival (52). Considering the limitation of WGS in clinical applications, the CHP score, which is based on transcript profiles, has potential use in predicting the prognosis of many types of tumors.

While our investigations have revealed novel associations of chromothripsis with tumor phenotypes, patient prognosis, and treatment efficacy, there important limitations of the current work worth pointing out. First, the CHP score does not perform well in predicting immunotherapy or prognosis of all tumor types, which indicates that more samples are needed to understand the heterogenous response of different tumor types. Second, the result of drug response in tumors with chromothripsis is based on cancer cell lines. More clinical trials are needed to verify the therapeutic utility of EGFR pathway inhibitors in cancer cases involving chromothripsis.

## Data availability statement

The datasets presented in this study can be found in online repositories. The names of the repository/repositories and accession number(s) can be found in the article/**Supplementary Material**.

## Author contributions

LY and QZ formal analysis, writing-original draft. HX and ZH: data retrive and curation. ZD and XK: visualization and formal analysis. ML and JZ: funding supporting. ML and YX: writing-review & editing. All authors contributed to the article and approved the submitted version.

## Supplementary material

Supplementary Figure 1 Flow chart of this study.

Supplementary Figure 2 KEGG enrichment analysis of top 20 enriched pathways between chromothripsis and non-chromothripsis groups.

Supplementary Figure 3 Correlation coefficients between the CHP score or specific immune signatures with responses to different treatments in the GSE194040 dataset after the correction of hormone receptor and human HER-2 factors.
