# Supplementary figures and images for: Pan-cancer analysis of chromothripsis-related gene expression patterns indicates an association with tumor immune and therapeutic agent responses
Source: Front Oncol. 2023 Jan 24;13:1074955. doi: 10.3389/fonc.2023.1074955 (PMC9902954; doi:10.3389/fonc.2023.1074955)

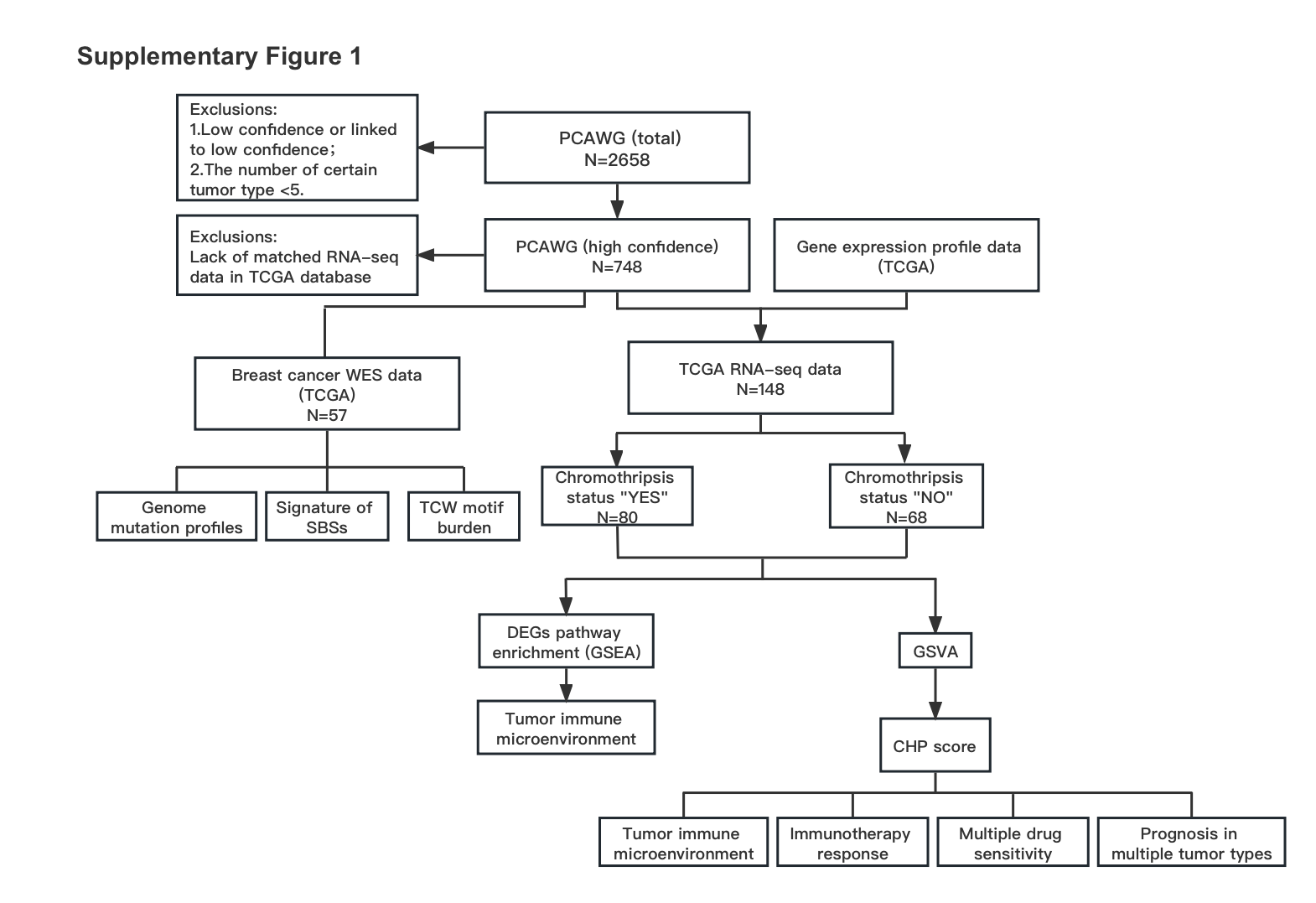

Supplement: Supplementary Figure 1 — Flow chart of this study. [file Image_1.jpeg]

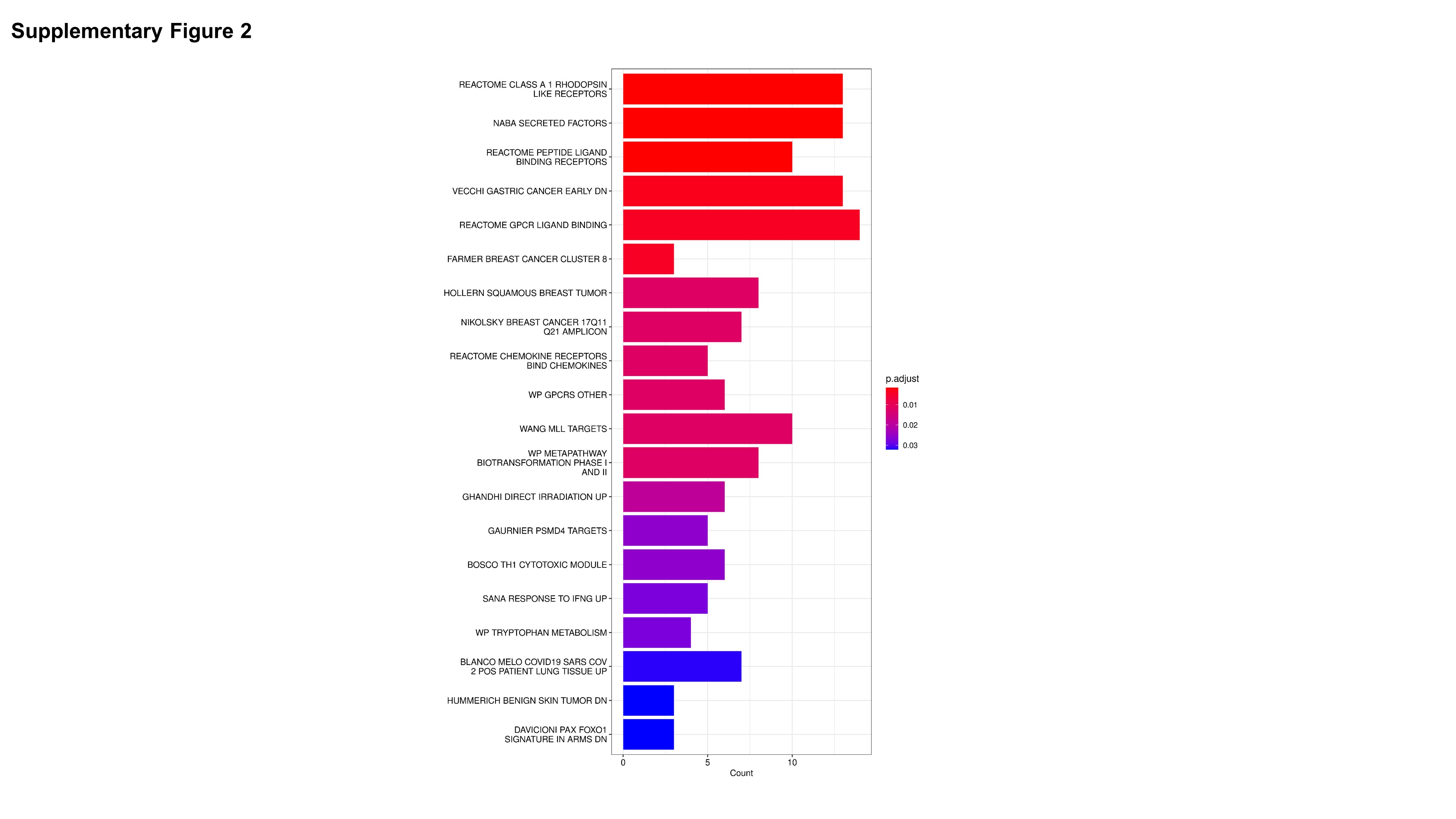

Supplement: Supplementary Figure 2 — KEGG enrichment analysis of top 20 enriched pathways between chromothripsis and non-chromothripsis groups. [file Image_2.jpeg]

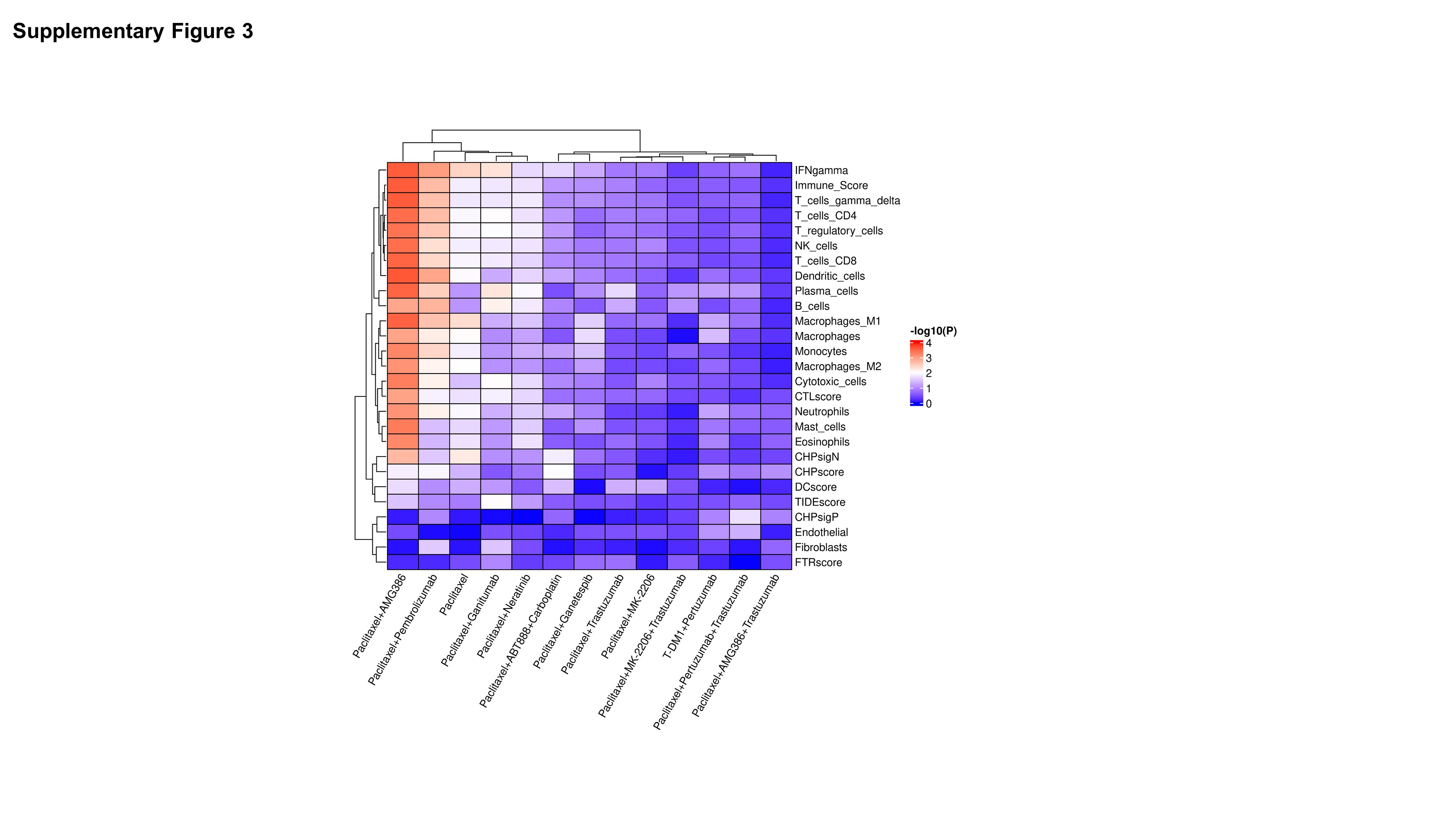

Supplement: Supplementary Figure 3 — Correlation coefficients between the CHP score or specific immune signatures with responses to different treatments in the GSE194040 dataset after the correction of hormone receptor and human HER-2 factors. [file Image_3.jpeg]
